# Killing of Targets by CD8^+^ T Cells in the Mouse Spleen Follows the Law of Mass Action

**DOI:** 10.1371/journal.pone.0015959

**Published:** 2011-01-24

**Authors:** Vitaly V. Ganusov, Daniel L. Barber, Rob J. De Boer

**Affiliations:** 1 Department of Microbiology, University of Tennessee, Knoxville, Tennessee, United States of America; 2 National Institutes of Health, Bethesda, Massachusetts, United States of America; 3 Theoretical Biology, Utrecht University, Utrecht, The Netherlands; Albert Einstein College of Medicine, United States of America

## Abstract

It has been difficult to correlate the quality of CD8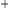 T cell responses with protection against viral infections. To investigate the relationship between efficacy and magnitude of T cell responses, we quantify the rate at which individual CD8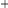 effector and memory T cells kill target cells in the mouse spleen. Using mathematical modeling, we analyze recent data on the loss of target cells pulsed with three different peptides from the mouse lymphocytic choriomeningitis virus (LCMV) in mouse spleens with varying numbers of epitope-specific CD8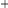 T cells. We find that the killing of targets follows the law of mass-action, i.e., the death rate of individual target cells remains proportional to the frequency (or the total number) of specific CD8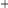 T cells in the spleen despite the fact that effector cell densities and effector to target ratios vary about a 1000-fold. The killing rate of LCMV-specific CD8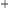 T cells is largely independent of T cell specificity and differentiation stage. Our results thus allow one to calculate the critical T cell concentration at which growth of a virus with a given replication rate can be prevented from the start of infection by memory CD8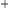 T cell response.

## Introduction

Vaccination is one of the most successful medical achievements of the last century. Due to our limited understanding of the correlates of protection, most vaccines have been developed by a trial and error approach, and we have yet failed to deliver vaccines for important diseases like AIDS or malaria. It is generally believed that most of the currently used vaccines provide protection by inducing high titers of pathogen-neutralizing antibodies [1]. The efficacy of an antibody-inducing vaccine tends to be proportional to the titer of neutralizing antibodies after vaccination [2]. The new vaccines that are currently being developed for devastating chronic infections, such as HIV and malaria, are designed to stimulate cellular CD4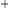 and CD8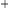 T cell responses. Such vaccines indeed elicit memory T cells, but at present it remains unclear whether or not this T cell memory can provide protection to infection, and which parameters of these T cells would correlate with protection [1]. It has been suggested that “polyfunctional” memory CD4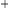 T cells, which produce a variety of different cytokines [3], are superior in providing protection to infection with Leishmania [4], and that polyfunctional memory CD8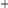 T cells are protective against SIV infection [5]. Evidence from HIV infected patients suggests that memory T cells loose functionality when viral loads are high [6], [7], arguing that the frequency of polyfunctional memory T cells is a consequence of the level of protection rather than its cause.

Our limited understanding of the level of protection provided by memory T cells is partly due to the fact that the functionality of effector and memory T cells is typically measured *in vitro*. There is very little quantitative data on the control of pathogen growth in tissues by T cell immunity [8], [9]. To quantify cytotoxic efficacy of CD8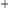 T cells *in vivo* one can transfer target cells that are pulsed with viral peptides into mice harboring virus-specific effector or memory CD8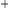 T cells, and follow the subsequent elimination of the pulsed target cells in the spleen [10]–[19]. We combine the data from recently published experiments [16] with a recently developed mathematical model [20]–[22] to quantify the rates at which individual effector or memory CD8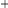 T cells kill target cells pulsed with epitopes from the mouse lymphocytic choriomeningitis virus (LCMV).

Even though the total number of LCMV-specific CD8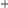 T cells (and the ratio of killers to targets) in the spleen varies almost 3 orders of magnitude in these experiments, we found that the death rate of peptide-pulsed targets due to CD8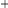 T cell mediated killing remains proportional to the frequency (or the total number) of virus-specific CD8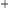 T cells in the spleen. Thus the *per capita* killing efficacy of CD8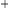 T cells, i.e., the average rate at which an individual CD8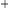 T cell kills targets, is largely independent of the density of the specific CD8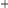 T cells. This suggests that vaccines increasing the number of virus-specific CD8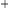 T cells, should proportionally increase the rate at which individual virus-infected cells are cleared by that T cell response. Using information on the rate of virus replication and the per cell killing efficacy of virus-specific CD8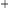 T cells one can calculate the critical number of memory CD8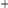 T cells required to control viral growth [23]. If these results can be generalized to other lymphoid and nonlymphoid organs and to other acute viral infections our results suggest that memory CD8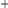 T cells are able to provide sterilizing immunity, if they are to be present at the right place and at high enough frequencies [24], [25].

## Materials and Methods

### Cytotoxicity in vivo

**Figure 1:**
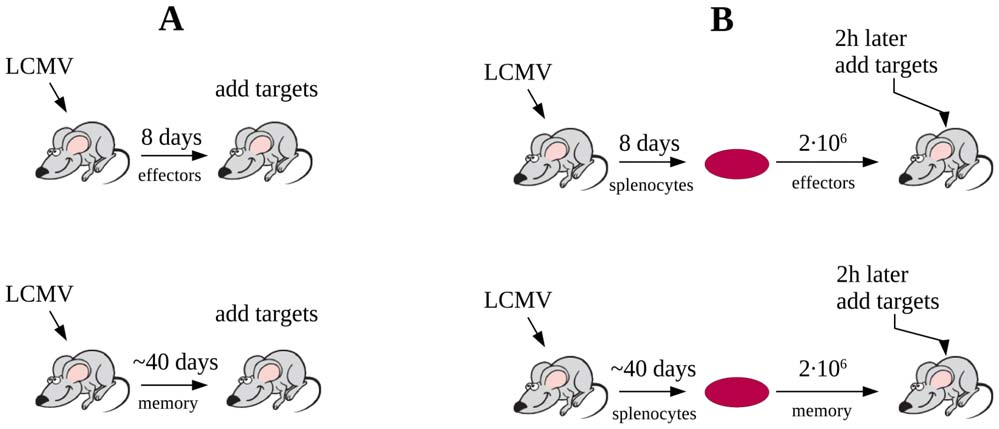
Schematic representation of the in vivo cytotoxicity assays undertaken to investigate the quantitative details of CD8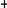 T cell mediated killing of peptide-pulsed targets in the mouse spleen. In the first set of experiments (“LCMV infection”, panel A), B6 mice were infected with LCMV-Arm and 8 or 37–100 days later, three populations of 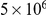 target cells (pulsed with either NP296 or GP276 peptides of LCMV and unpulsed) were transferred into these mice. In the second set of experiments (“adoptive transfer”, panel B), P14 TCR Tg CD8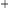 T cells, specific to the GP33 epitope of LCMV, were transferred into B6 mice and then infected with LCMV-Arm. Eight or 40 days later, different number of effector (day 8) or memory (day 40) P14 CD8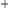 T cells from these mice were transferred into new naive B6 mice (in panel B, we shown an example where 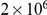 effectors or memory CD8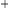 T cells are transferred). Two hours later, two populations of 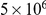 targets (pulsed with the GP33 peptide of LCMV and unpulsed) were transferred into these mice now harboring GP33-specific CD8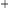 T cells. In both sets of experiments, killing of peptide-pulsed targets was measured in spleens of mice at different times after cell transfer [16].

We analyze published data on killing of peptide-pulsed splenocytes by LCMV-specific effector and memory CD8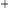 T cells [16]. The experimental method of measuring cytotoxicity of CD8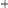 T cells in vivo has been described in detail elsewhere [19], and the reader is referred to the original publications for more detail. In the first set of experiments (“in vivo LCMV infection”), target splenocytes were pulsed with NP396 or GP276 peptides of LCMV (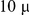
M) or left unpulsed. Targets were subsequently transferred into syngenic mice either infected with LCMV 8 days previously (“acutely infected” mice) or recovered from LCMV infection (LCMV-immune or “memory” mice). At different times after the transfer of targets, spleens were harvested, and the number of pulsed and unpulsed targets, splenocytes, and peptide-specific CD8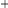 T cells was calculated (Fig. 1 A).

In the second set of experiments (“adoptive transfer”), 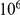 P14 CD8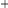 T cells, expressing a TCR specific for the GP33 epitope of LCMV, were adoptively transferred into recipient B6 mice which were then infected i.p. with LCMV-Arm [26]. Eight (for effectors) or 40 (for memory T cells) days later, different numbers of P14 CD8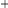 T cells harvested from these mice were transferred into new naive recipients (Fig. 1B). The number of effector CD8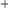 T cells transferred into different recipients was 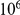, 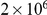, 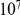, and 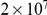. The number of memory CD8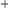 T cells transferred into different recipients was 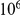, 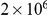, and 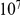. Two hours later, two populations of CFSE labeled splenocytes, one of which was pulsed with the GP33 peptide of LCMV (1 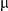
M), were transferred into these recipient mice, harboring the transferred GP33-specific effector or memory CD8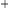 T cells. The percent of targets killed was calculated at different times after target cell transfer (as described earlier [16], [19]). The ratio of the frequencies of peptide-pulsed and unpulsed targets was used in fitting of the data and was calculated as 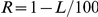, where 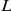 is the percent of peptide-pulsed targets killed [19], [22].

### Mathematical model for the cytotoxicity in vivo assay

Details of the mathematical model proposed to describe migration of injected targets from the blood to the spleen and killing of peptide-pulsed targets in the spleen are given elsewhere [20], [ 22, see also Supporting Information]. In short, target cells that are injected i.v., migrate from the blood to the spleen at a rate 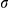, die at a rate 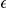 due to preparation techniques (independent of CD8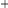 T cell mediated killing), or migrate to other tissues and/or die elsewhere at a rate 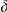. In the spleen, targets die due to preparation-induced death rate 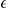, and peptide-pulsed targets also die due to CD8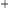 T cell mediated killing, described by the rate 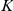. The dynamics of the total number of unpulsed targets in the spleen 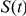 and the ratio of the frequency of peptide-pulsed to unpulsed targets in the spleen 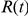 was previously [22] shown to be
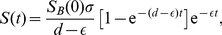
(1)

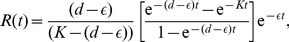
(2)where 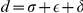 is the rate of removal of cells from the blood and 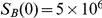 is the initial number of unpulsed targets in the blood [16].

We have shown previously that the rate of recruitment of target cells from the blood to the spleen depends on the size of the spleen [22]. Therefore, to describe recruitment of targets into the spleen we let the rate of recruitment be 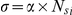 where 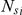 is the number of splenocytes in the 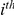 mouse and 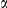 is a coefficient [22]. It should be emphasized, however, that our conclusions are not changed if we assume a fixed rate of recruitment of targets from the blood to the spleen (results not shown). By estimating the death rate of peptide-pulsed targets we avoided the problem of unintended variation in the frequency (or number) of peptide-specific CD8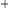 T cells in individual mice [22]. This variation in the frequency of epitope-specific CD8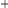 T cells in spleens of identically treated individual mice could be biologically relevant, and hence influence the killing rate, or could represent measurement noise. The absence of a positive correlation between the number of targets killed and the CD8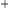 T cell frequency in identically treated mice suggests that the variation in this frequency of epitope-specific CD8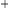 T cells is due to measurement noise (see Figs. S1 and S2 in Supporting Information S1).

To fit the data on recruitment of targets into the spleen and on killing of peptide-pulsed targets in the spleen simultaneously, we 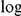-transform the data and the model predictions. To access lack of fit of the data with repeated measurements we use the F-test [27]. Fittings were done in Mathematica 5.2 using the routine FindMinimum. Confidence intervals for parameters were calculated by bootstrapping the data with 1000 simulations [51].

### Mathematical model for the virus dynamics

To describe the effect of the initial number of virus-specific CD8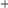 T cells on the virus dynamics, we formulate the following mathematical model. In the absence of the CD8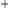 T cell response the virus population expands exponentially at the rate 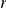 from the initial inoculum 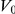 and can potentially reach a maximum density 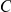 (carrying capacity). CD8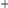 T cell response starts with 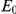 precursors and follows a programmed response [28]–[30]. CD8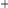 T cells become activated at time 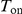 and the population expands at the rate 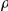. At time 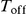 the response stops. Virus-specific CD8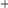 T cell response clears the virus at the rate proportional to the product of the density of the virus and CD8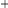 T cell response. The dynamics of the virus and virus-specific CD8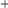 T cell response is thus given by the following equations:
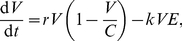
(3)

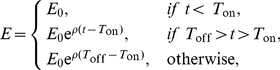
(4)where 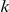 is the killing efficacy of T cells, and 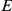 is the frequency (or number) of virus-specific CD8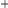 T cells. Because in the model the virus is generally cleared before the peak of the CD8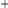 T cell response we do not model contraction and memory phases for the virus-specific CD8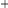 T cells.

## Results

### Death rate of targets following LCMV infection

To quantify the rates at which effector and memory CD8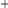 T cells kill their targets in a mouse spleen, we have previously analyzed data from recently published experiments on the killing of targets pulsed with either NP396 or GP276 peptides from LCMV by peptide-specific effector or memory CD8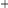 T cells [16], [22]. Mice, infected with LCMV-Armstrong develop a vigorous CD8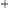 T cell response that peaks 8 days after the infection [31], [32]. By 15–30 days after the infection, most of effectors die and a population of LCMV-specific memory CD8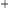 T cells persists for the life of the animal [32]. We have recently extended a mathematical model to estimate the average death rate of target cells in such a *in vivo* cytotoxicity assay [22]. The extended model describes the recruitment of target cells from the blood to the spleen, non-specific death of targets (e.g., due to the experimental preparation), and killing of peptide-pulsed targets by peptide-specific CD8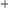 T cells in the spleen [22]. By fitting this model to the data we previously estimated the average death rate of peptide-pulsed targets due to killing by the effector or memory CD8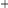 T cell response (Table 1 in Supporting Information). Not surprisingly, the death rate of targets was highly correlated with the magnitude of the epitope-specific CD8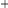 T cell response [22]. The immunodominant NP396-specific effector CD8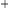 T cell response was most efficient at killing targets, while a smaller population, i.e., GP276-specific memory CD8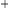 T cells, induced the slowest rate of killing (see Fig. 3 and Table S1 in Supporting Information S1).

### Death rate of targets following adoptive transfer

**Figure 2:**
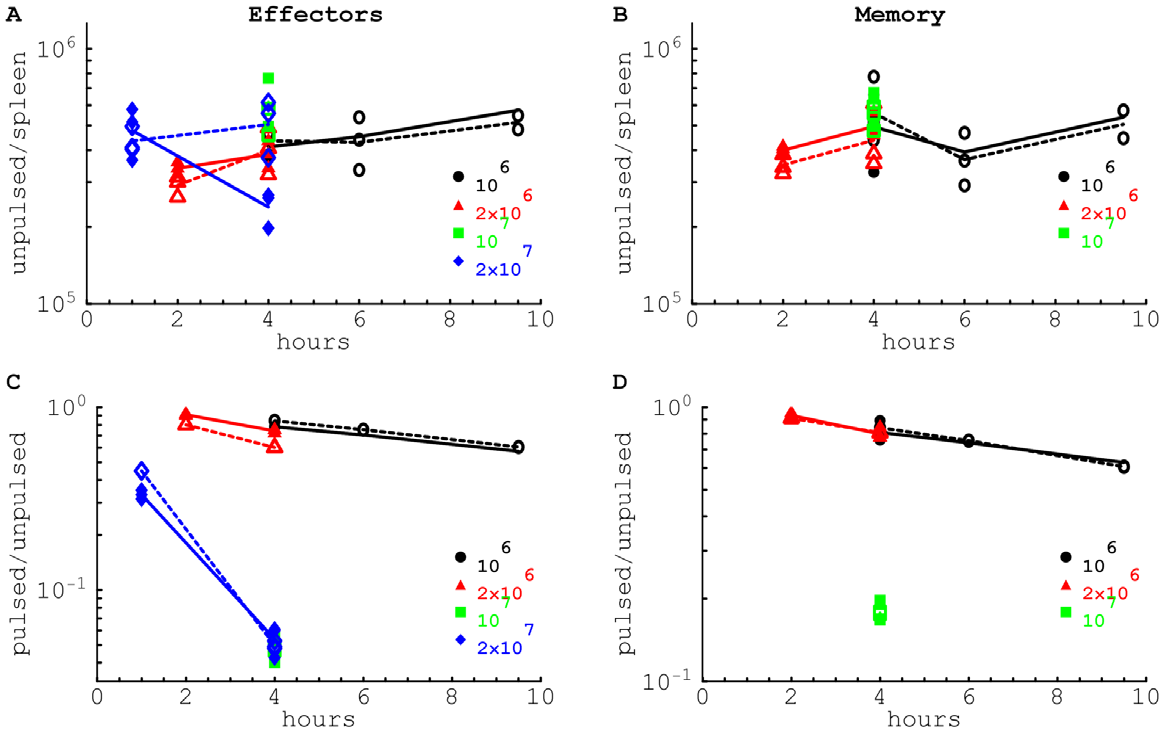
Fits of the mathematical model to data involving adoptive transfer of different numbers of GP33-specific effector (panels A&C) or memory (panels B&D) CD8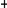 T cells. Panels A and B show that the number of unpulsed targets in the spleen at different times after cell transfer remains approximately constant. Panels C and D show the decrease in the ratio of the frequencies of peptide-pulsed and unpulsed targets in the spleen over time. Different symbols denote data from different adoptive transfer experiments with 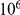, 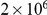, 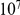, or 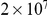 P14 cells transferred. Filled symbols denote individual mouse measurements with the averages per time point being connected by solid lines. Open symbols are the model predictions with averages being connected by dashed lines. Parameters providing the best fits of the model are shown in Table 1. Note that in panel A, the model can not predict the decline in the number of unpulsed targets with time in experiments with transfer of 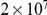 GP33-specific effectors. Such decline in the number of unpulsed targets in the spleen is unexpected and is most likely due to a measurement error.

To further investigate the relationship between the death rate of peptide-pulsed targets and the frequency (or the number) of epitope-specific CD8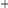 T cells in the spleen, we secondly analyzed data from adoptive transfer experiments, involving the transfer of different numbers of effector or memory CD8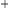 T cells specific for the GP33 epitope of LCMV [16]. Approximately 2 to 10% of the adoptively transferred CD8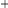 T cells accumulated in the mouse spleen (Table 1). Transfer of different numbers of epitope-specific CD8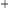 T cells led to markedly different frequencies and numbers of these cells in the spleen, and as a consequence, to markedly different effector to target ratios (Table 1). Two hours after the transfer of CD8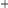 T cells, GP33-pulsed and unpulsed target cells were transferred into the same mice, and killing of peptide-pulsed targets was measured at different time points (Fig. 2).

**Table 1:** Estimates of parameters of the mathematical model fitted to the data from the adoptive transfer experiments.

Parameter	Mean	95% CIs	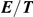	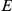 , %	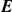 , 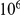 cells	Cellstransferred
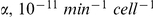	1.93	1.67– 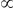				
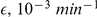	1.35	0.28–1.97				
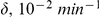	0.79	0.52– 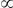				
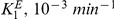	1.28	0.96–1.50	0.14	0.06	0.05	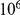
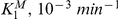	1.08	0.81–3.83	0.09	0.04	0.03	
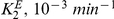	1.83	1.06–2.05	0.34	0.18	0.10	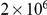
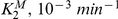	1.35	0.76–1.59	0.25	0.15	0.09	
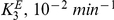	3.23	1.27–4.48	23.7	0.87	0.68	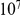
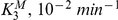	1.29	0.72–1.51	9.48	1.25	1.05	
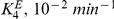	3.20	1.21–3.96	44.4	1.54	1.43	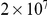

In different experiments, 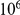, 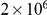, 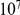 or 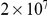 effector or memory CD8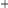 T cells were transferred resulting in the shown average effector to target ratio 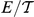, average percentage or the total number of transferred cells in spleens of the recipient mice. We estimated the death rate of peptide-pulsed targets due to killing by effector (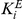) or memory (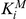) CD8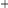 T cells for different numbers of CD8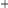 T cells transferred. Since the first measurements in these experimental data were taken 1 hour after transfer of target cells, we obtained unbound confidence intervals for the parameters 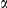 and 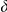 that determine the rate of recruitment of target cells from the blood to the spleen and elsewhere, respectively. Interestingly, to properly describe these data we require the preparation-induced cell death rate that we have previously postulated to exist [22], although in these experiments this rate was smaller than that during acute LCMV infection (see Table 2 in Supporting Information). Note that in these experiments the effector to target ratio 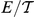 changes over 100 fold (from 0.1 to over 10).

**Figure 3:**
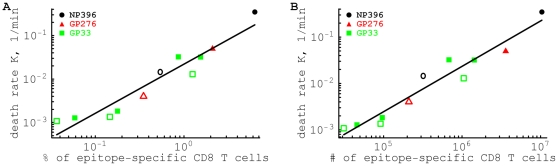
The estimated death rate of peptide-pulsed targets due to killing by epitope-specific CD8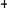 T cells is proportional to the average percent (panel A) or average number (panel B) of epitope-specific CD8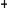 T cells in the spleen. Note that the percent of antigen-specific CD8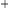 T cells is calculated among all cells in the spleen. Estimates are given for targets pulsed with NP396 (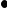), GP276 (▴), or GP33 (▪) peptides from LCMV. Filled symbols are for killing by effector CD8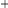 T cells, and open symbols are for killing by memory CD8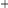 T cells. Lines show the linear regression for the 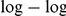 transformed estimates of the death rate and density of CD8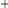 T cells. Slopes for the regressions are not statistically different from one (panel A: slope = 1.13, 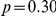; panel B: slope = 0.98, 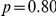).

To estimate the average death rate of GP33-pulsed targets due to the killing by GP33-specific effector or memory CD8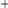 T cells, we fitted the same model (Eqs. 1–2) to these data (Table 1). The model described the data very well with the exception of one time point where very few unpulsed targets were recruited into the spleen (Fig. 2A at 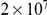 effector CD8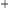 T cell transferred; lack of fit test with this time point removed: 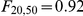, 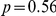). As expected, there was a strong correlation between the number of GP33-specific CD8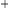 T cells that were transferred and the estimated death rate of peptide-pulsed targets (Table 1 and Fig. 3).

### Killing *in vivo* follows the law of mass-action

**Figure 4:**
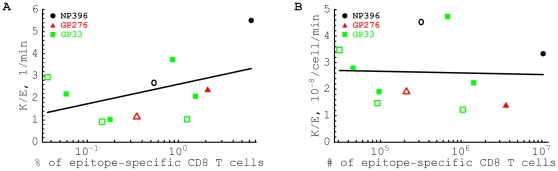
The estimated per capita killing efficacy of CD8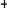 T cells is independent of the average percent (panel A) or average number of epitope-specific CD8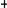 T cells in the spleen. We estimate the killing efficacy 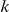 by dividing the death rate of targets 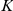 by the frequency (panel A) or the number (panel B) of epitope-specific CD8 T cells in the spleen 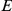. Notations are the same as in Fig. 3. Lines show linear regression for the estimates of the killing efficacy and 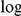 transformed frequency (panel A) or number (panel B) of epitope-specific CD8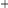 T cells. Slopes for the regressions are not statistically different from zero (panel A: slope = 0.89, 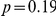; panel B: slope = −0.06, 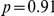). A large positive slope for the correlation between per capita killing 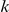 and the frequency of epitope-specific CD8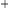 T cells in panel A is due to an outlier for killing by NP396-specific effector CD8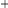 T cells. Removing this outlier led to the estimated slope = 0.06, 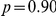. The average killing efficacy of CD8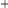 T cells is 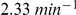 (panel A) or 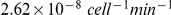 (panel B).

From these two types of experiments, we have in total 11 estimates for the average death rate of target cells elicited by LCMV-specific CD8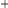 T cells, present at frequencies that vary a 100-fold or numbers that vary almost a 1000-fold (Tables 1 and 2 in the Supporting Information). We find a strong positive correlation between the average death rate of targets 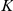 and the average frequency (Fig. 3A), or the average number (Fig. 3B), of epitope-specific CD8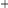 T cells in the mouse spleen. Importantly, the slope of the 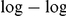 correlation was not significantly different from one, suggesting that the death rate of peptide-pulsed targets is simply proportional to the frequency, or the total number, of epitope-specific CD8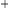 T cells in the spleen, i.e., 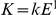. This linear dependence of the death rate of targets on the specific CD8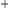 T cells was confirmed by normalizing the death rate of pulsed targets 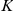 by the frequency (or the number) of epitope-specific CD8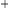 T cells in the spleen (i.e., by plotting 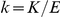 in Fig. 4). This constant level of the *per capita* killing efficacy of CD8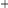 T cells 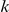 was observed over a 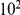 fold changes in the frequency and almost 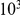 fold change in the number of epitope-specific CD8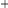 T cells. These results suggest that killing of target cells pulsed with LCMV peptides, by LCMV-specific CD8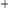 T cells conforms to the law of mass-action, whereby the death rate of targets remains proportional to the frequency (or the number) of epitope-specific CD8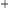 T cells [23], [33].

### Predicting the number of memory CD8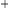 T cells needed for protection

**Figure 5:**
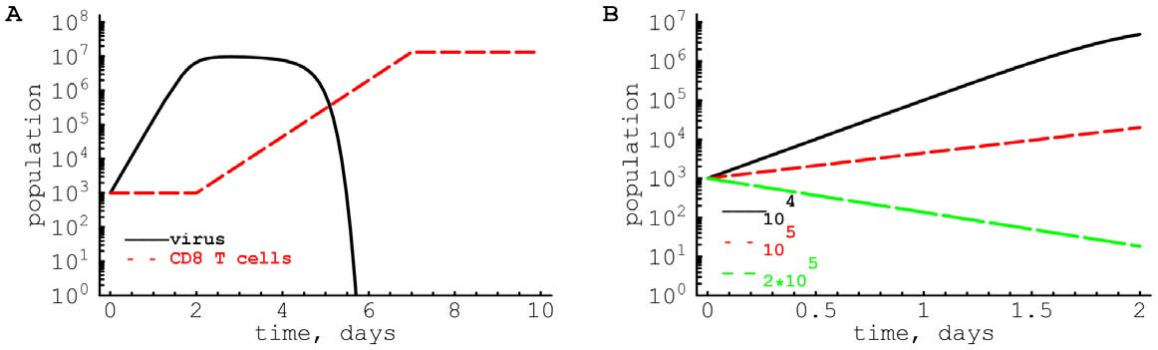
Predicted changes in viral load for different initial numbers of virus-specific memory CD8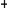 T cells. Using the model given in eqns. (3)–(4), we plot the dynamics of the virus (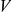) over the course of infection when the initial number of virus-specific memory CD8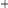 T cells (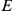) is low (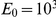, panel A) or high (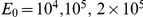, panel B). Viral density declines following infection as memory CD8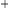 T cells reach the threshold level of 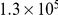 cells per spleen. Other parameters are 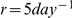, 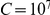, 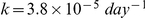, 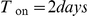, 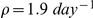, 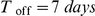 [29].

Our result on mass-action killing allows for a simple estimate of the level of virus-specific memory CD8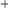 T cells that is required to provide protection to a subsequent viral infection [23]. We formulate a mathematical model that describes the dynamics of the virus and virus-specific CD8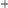 T cell response (see eqns. (3)–(4) in Materials and Methods). When the initial number of virus-specific CD8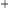 T cells is low, the virus grows until reaches carrying capacity and then is cleared by the expanded CD8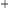 T cell response (Fig. 5A). However, if the initial number of virus-specific memory CD8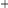 T cells is large enough, viral population declines after the infection (Fig. 5B). In the model, to prevent viral growth one requires that 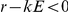, implying that the level of memory cells 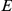 should exceed 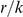. For instance, for LCMV-Armstrong with an initial replication rate of 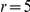 per day [34]–[36], and an estimated killing rate of 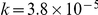 per cell per day (Fig. 4), we would predict that 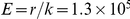 memory CD8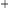 T cells per spleen should be able to provide sterilizing immunity against LCMV-Armstrong (Fig. 5B). The major difficulty with testing this prediction is that one needs to know the number, and/or the recruitment, of cytotoxic effector cells in the organ in which the virus is initially replicating. For the case of LCMV-Armstrong replicating mainly in the spleen after intraperitoneal infection, the density of LCMV-specific memory T cells in the spleen should be a strong predictor of the protection against the infection. It is interesting to note that it was previously found that 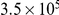 LCMV-specific memory CD8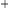 T cells are sufficient to clear chronic LCMV clone 13 infection [37] which is close to our estimate of 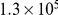 required to clear LCMV-Armstrong.

For viruses infecting other organs (e.g., flu infecting lung epithelial cells) predicting the number of CD8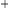 T cells required for protection may be more difficult because one has to estimate accumulation of effector/memory cells in that organ following reinfection (e.g., lung). The efficacy of virus-specific CD8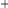 T cells at killing targets in peripheral organs could be different from that in the spleen and this difference could further complicate the prediction of the number of memory CD8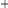 T cells required for protection.

Our results confirm and extend recent findings on killing of peptide-pulsed targets by effector CD8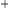 T cells specific to ovalbumin in collagen-fibrin gels in vitro [23]. The authors also suggested that killing of targets in gels also follows the law of mass-action with the death rate of targets increasing with CD8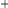 T cell concentration, although the range of T cell concentrations at which this dependence was seen was much smaller (about 4–10 fold). There are also quantitative differences: we find much faster killing rates (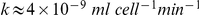 in vivo assuming that spleen volume is 0.15 ml [23], vs. 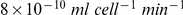 found in gels). This difference could be due to intrinsic differences in killing in vitro and in vivo, due to different ways of obtaining activated CD8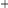 T cells, or due to artifacts in the in vivo cytotoxicity assay (see Discussion). Our current results support the notion that for a given viral infection there is a critical T cell concentration at which viral growth can be suppressed (Fig. 5) providing support for the development of sterilizing T cell-based vaccines [24], [25].

## Discussion

Recent interest in T cell based vaccines against several chronic infections of humans requires the development of experimental and theoretical tools to access the efficacy of such vaccines [38], [39]. It is generally believed that memory CD8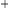 T cells induced by vaccination are not able to provide sterilizing immunity, because T cells react only to infected cells, i.e., after the infection has been established, and because CD8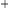 effector T cells operate at relatively high effector∶target ratios [39]. However, a recent study has shown that memory CD8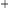 T cells can provide sterilizing immunity against malaria in mice; although for protection a very large population of memory cells was required [24]. Similarly, protection to small doses of SIV was observed in monkeys only if large and active populations of specific CD8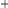 T cells were maintained by a chronic infection continuously expressing SIV epitopes [25].

Quantitative approaches aimed at estimating the *in vivo* efficacy of effector and memory CD8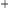 T cells, and the effects of CD8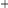 T cell densities on the control of pathogens, help to understand what densities need to be induced by T cell-based vaccines, and whether or not this is feasible. By fitting mathematical models to *in vivo* data we found that the death rate of targets due to killing by LCMV-specific CD8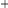 T cells is simply proportional to the average frequency (or number) of epitope-specific CD8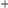 T cells in the mouse spleen (Fig. 4). This is a surprising result because in these experiments the frequencies of LCMV-specific CD8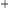 T cells in the spleen vary about a 100-fold (from 0.06% to 6%), their numbers vary almost a 1000-fold, and the killer to target ratio varies over 1000 fold. Technically this means that the killing of targets by CD8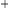 T cells follows the law of mass action [23], [33], [40], and that CTLs do not compete for access to targets whenever their frequencies in the spleen remain below 6% of the splenocytes (or their numbers below 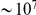 cells). From basic principles of cell interactions one does expect that the death rate of targets should at some point saturate when the density of killer CD8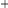 T cells increases [41]–[44]. In particular, it was found in *in vitro* experiments that the death rate of targets *in vitro* bound by 2 CTL is not dramatically different from death rate of targets bound by 3 or 4 CTLs [42]. Furthermore, in *in vitro* experiments involving the 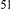
Cr release assay, killing often saturates at reasonably small E/T ratios (e.g., in [45] at E/T around 10). In our in vivo experiments, E/T ratio varied from 0.1 (adoptive transfer experiments) to over 100 (endogenous response to LCMV). Therefore, it is surprising that *in vivo* the death rate of targets remains simply proportional to the density of killers. Possible explanations for the lack of a saturation in the death rate with CD8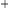 T cell density in these *in vivo* data vary from experimental artifacts to biological reality.

First, the observed high densities of epitope-specific CD8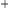 T cells in the spleen could still be too low to cause saturation in the death rate of targets. At the peak of the immune response to LCMV, NP396-specific CD8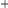 T cells are the most abundant T cell population in the spleen, and yet this population constitutes only 6% of all splenocytes, which remains a fairly small fraction. If saturation only occurs at even higher densities, our results would be generic and killing would follow mass-action kinetics for realistic effector cell densities.

Second, the mass-action could be an artifact of the experimental procedure because the loss rate of target cells was only measured after making a single cell suspension from the spleen, and sorting the cells by their CFSE fluorescence. This experimental procedure, and the time delay between the *in vivo* encounter between the target cell and its specific killer cell, may give the targets ample time to die after a brief, and perhaps marginal exposure, to a specific CD8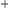 T cell. Thus, it seems possible that in this *in vivo* cytotoxicity assay largely measures the initial rate of encounter between target cells and CD8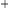 T cells, and hardly the rate of killing of targets. Such initial encounter rates would *a priori* be expected to follow mass-action kinetics. Our estimated half-life of NP396 pulsed target cells is about three minutes [22], which is much shorter than the 10 to 60 minutes it takes a target cell to die after a contact with the CTL *in vitro* or *in vivo* [42], [46], [47]. At high effector to target ratios the three minutes time might be sufficient to have an encounter between most of targets and an effector cell, and possibly to have some perforin molecules being delivered to the target cell. Such an encounter need not lead to to immediate death of the target cells *in vivo*, but their subsequent experimental manipulation *in vitro* could lead to cell death at the time the cell suspensions are produced. If this is true, we are overestimating the death rate, and if prolonged contacts between effectors and targets are required for cell death *in vivo*, we may be underestimating the saturation effects.

The possibility that preparation of cell suspensions could lead to an early death of peptide-pulsed targets is corroborated by recent work demonstrating that granzymes A and B are dispensable for the killing of peptide-pulsed targets by CD8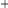 T cells or NK cells in vivo, despite its importance in the control of viral infections [48]. In the experiments, an initial delivery of perforin might suffice to induce cell death during the experimental preparation of the cells.

Finally, it is possible that our results arise due to the fact that we combine data on the killing by CD8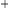 T cells of different specificities. It has been proposed that the *per capita* killing efficacy of NP396-specific CD8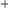 T cells is higher than that of GP276-specific CD8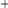 T cells [20], [21]. However, since the *per capita* killing efficacy of CD8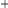 T cells of different specificities varies only 2–5 fold (Fig. 4), while the CD8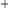 T cell frequencies in the spleen vary more than two orders of magnitude, this seems an unlikely explanation.

A recent study re-analyzing the same published data suggested that killing of targets saturates when the frequency of LCMV-specific CD8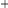 T cells breaches 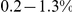 in the spleen [44]. This is in disagreement with the range of frequencies that we find consistent with mass-action killing. The contradiction may be due to the different ranges in frequencies used to analyze the data. In the previous study, the variation in frequencies of epitope-specific CD8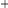 T cells in individual mice was considered to be correlated with killing of targets in these mice. This variation in frequency of a given epitope-specific response was in general relatively small, however. At such small scales evidence for saturation can come about from noise in the measurements of CD8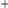 T cell frequencies in spleens of individual mice (Ganusov and De Boer, ms. in preparation). In our current analysis we focused on a large variation in CD8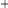 T cell frequencies/numbers between different epitopes (100 to 1000 fold) and therefore we believe that our results are more reliable.

It remains unclear whether the killing of targets depends on the frequency or on the total number of epitope-specific CD8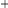 T cells in the spleen [22]. We find mass-action kinetics for both cases (Fig. 4). To estimate whether individual effector CD8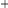 T cells are more efficient killers than individual memory CD8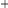 T cells, one needs to know whether killing depends on cell frequencies or on cell numbers. Memory NP396- and GP276-specific CD8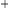 T cells are only half as efficient killers as effectors of the same specificity when killing is considered to be proportional to cellular frequencies (Fig. 4A and [21]). In contrast, if killing depends on cell numbers, individual NP396- and GP276-specific memory T cells are more efficient killers than the corresponding effector cells (Fig. 4B). Understanding whether killing frequency- or number-dependent is critical for prediction of the efficacy of T-cell based vaccines, in part, because the number and the frequency of memory T cells are affected differently following infections with heterologous viruses [49], [50]. If killing indeed generally obeys mass-action kinetics one should be able to generalize the calculations of the critical T cell concentration to other viruses [23], and this theoretical framework could therefore provide guidelines for estimating the efficacy of T-cell based vaccines.

## Supporting Information

Supporting Information S1Here we show a mathematical model for the in vivo cytotoxicity assay, estimates of the death rate of targets pulsed with NP396 and GP276 peptides following acute LCMV infection and the correlations between the fraction of target cells killed and the frequency of epitope-specific CD8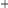 T cells in the spleen.(PDF)
