# Supplementary material for: Killing of Targets by CD8+ T Cells in the Mouse Spleen Follows the Law of Mass Action
Source: PLoS One. 2011 Jan 24;6(1):e15959. doi: 10.1371/journal.pone.0015959 (PMC3025913; doi:10.1371/journal.pone.0015959)
Supplement: Supporting Information S1 — Here we show a mathematical model for the in vivo cytotoxicity assay, estimates of the death rate of targets pulsed with NP396 and GP276 peptides following acute LCMV infection and the correlations between the fraction of target cells killed and the frequency of epitope-specific CD8 T cells in the spleen. (PDF) [file pone.0015959.s001.pdf]

Supporting Information S1

Mathematical model for the in vivo cytotoxicity assay

The dynamics of unpulsed and peptide-pulsed targets in the blood and in the spleen are given by equations

$$\frac{\mathrm{d}S_B(t)}{\mathrm{d}t} = -(\delta+\sigma+\epsilon)S_B(t), \tag{A.1}$$

$$\frac{\mathrm{d}S(t)}{\mathrm{d}t} = \sigma S_B(t) - \epsilon S(t), \tag{A.2}$$

$$\frac{\mathrm{d}T_B(t)}{\mathrm{d}t} = -(\delta+\sigma+\epsilon)T_B(t), \tag{A.3}$$

$$\frac{\mathrm{d}T(t)}{\mathrm{d}t} = \sigma T_B(t) - \epsilon T(t) - KT(t), \tag{A.4}$$

where $S_B(t)$ and $T_B(t)$ are the numbers of unpulsed and peptide-pulsed target cells in the blood, respectively, and $S(t)$ and $T(t)$ are the numbers of unpulsed and pulsed targets in the spleen, respectively, σ is the rate of migration of target cells from the blood into the spleen, and δ is the rate of cell migration/death from blood to other organs, ϵ is the extra death rate of transferred splenocytes due to experimental preparation (independent of epitope-specific $CD8^+$ T cells), and K is the death rate of peptide-pulsed targets due to $CD8^+$ T cell mediated killing in the spleen. For our experiments, the initial conditions for the model are $S_B(0) = T_B(0) = 5 \times 10^6$ cells and $S(0) = T(0) = 0$ (16). In the case where all model parameters, including the death rate of targets due to $CD8^+$ T cell mediated killing, are independent of time, the model can be solved analytically (22); and the particular solution for K is shown in eqn. (1) and (2) in the main text.

Parameter	Mean	95% CIs	$E/\mathcal{T}$	E, %	E, 10^6 cells	cell type
$\alpha_A,\ 10^{-12}\ min^{-1}$	7.17	$5.63 - 9.25$				
$\alpha_M,\ 10^{-11}\ min^{-1}$	1.30	$0.90 - 1.88$				
$\epsilon,\ 10^{-3}\ min^{-1}$	4.71	$3.37 - 6.12$				
$K^E_{NP},\ min^{-1}$	3.45×10^{-1}	$(2.59 - 4.56) \times 10^{-1}$	2471	6.3	10.4	NP396-spec effectors
$K^E_{GP},\ min^{-1}$	5.0×10^{-2}	$(3.80 - 6.41) \times 10^{-2}$	130	2.1	3.6	GP276-spec effectors
$K^M_{NP},\ min^{-1}$	1.44×10^{-2}	$(1.10 - 1.82) \times 10^{-2}$	4.9	0.54	0.32	NP396-spec memory
$K^M_{GP},\ min^{-1}$	4.15×10^{-3}	$(3.21 - 5.52) \times 10^{-3}$	1.5	0.35	0.21	GP276-spec memory

Table S1: Parameters providing the best fit of the mathematical model fitted to data on killing of NP396- and GP276-pulsed targets by LCMV-specific effector and memory CD8$^+$ T cells. Note that this table repeats estimates of the death rate of peptide-pulsed targets from our previous work (22). The table was extended to include values for the average effector to peptide-pulsed target ratio ($E/\mathcal{T}$), the average percentage, and the average total number of epitope-specific CD8$^+$ T in the spleen obtained from the data. Here α_A and α_M are coefficients relating the recruitment rate of cells into the spleen $\sigma = \alpha N_{si}$ in acutely infected (α_A) and memory (α_M) mice, and N_{si} is the number of splenocytes in individual mice,, K^E_{NP} and K^E_{GP} are the death rates of NP396- and GP276-pulsed targets due to killing by epitope-specific effectors, K^M_{NP} and K^M_{GP} the death rates of NP396- and GP276-pulsed targets due to killing by epitope-specific memory T cells. In the fits the rate of migration of labeled splenocytes to other organs δ was fixed to 0 since this did not affect the quality of the model fit to data (F-test for nested models: $F_{1,191} = 0.15$, $p = 0.70$). CIs were calculated by bootstrapping the data with 1000 simulations (51).

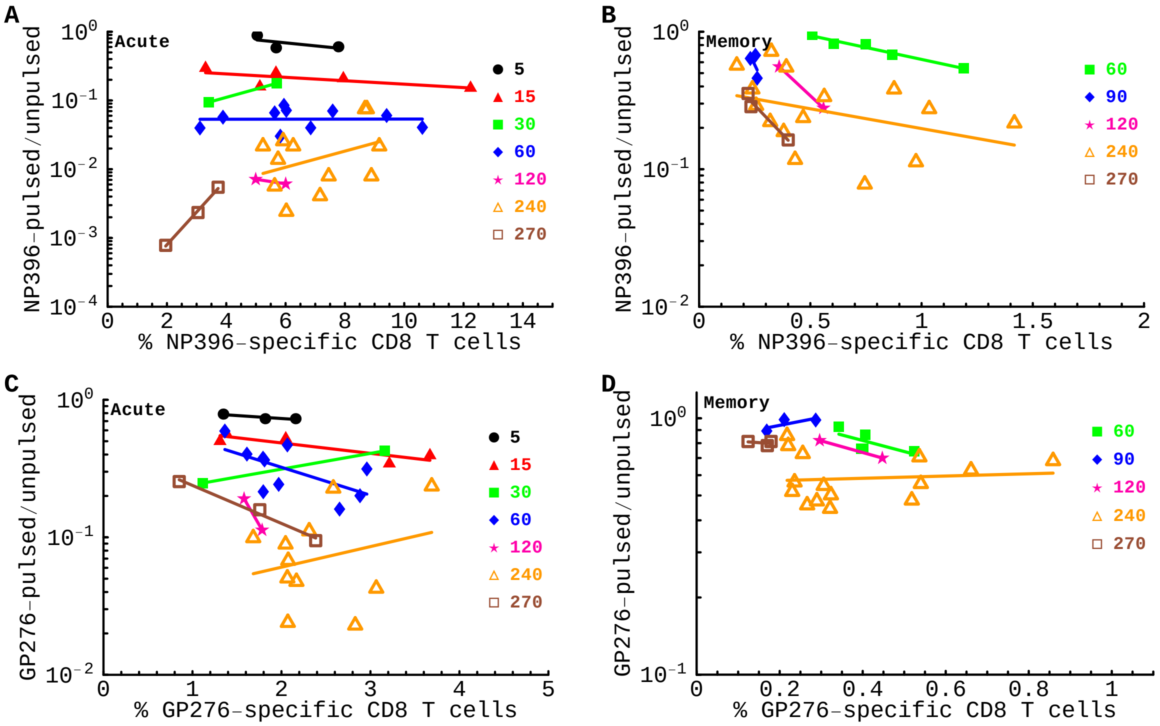

Figure S1: The absence of a significant negative correlation between killing of targets (measured as the ratio R) and the percent of epitope-specific $CD8^+$ T cells in the mouse spleen in acutely infected (panels A and C) and LCMV-immune (panels B and D) mice. For each time point in a group of identically treated mice, we test whether the unintentional variation in the density of effector cells is correlated with the loss rate of pulsed target cells. To visualize the data, we use different scales on the plots. Correlations tend to be weak and sometimes in the unexpected direction, e.g., in acutely infected mice (panels A and C), we find positive correlations between the persistence of pulsed targets and the frequency of peptide-specific $CD8^+$ T cells. A similar result is obtained if instead of the frequency of $CD8^+$ T cells we used total number (results not shown).

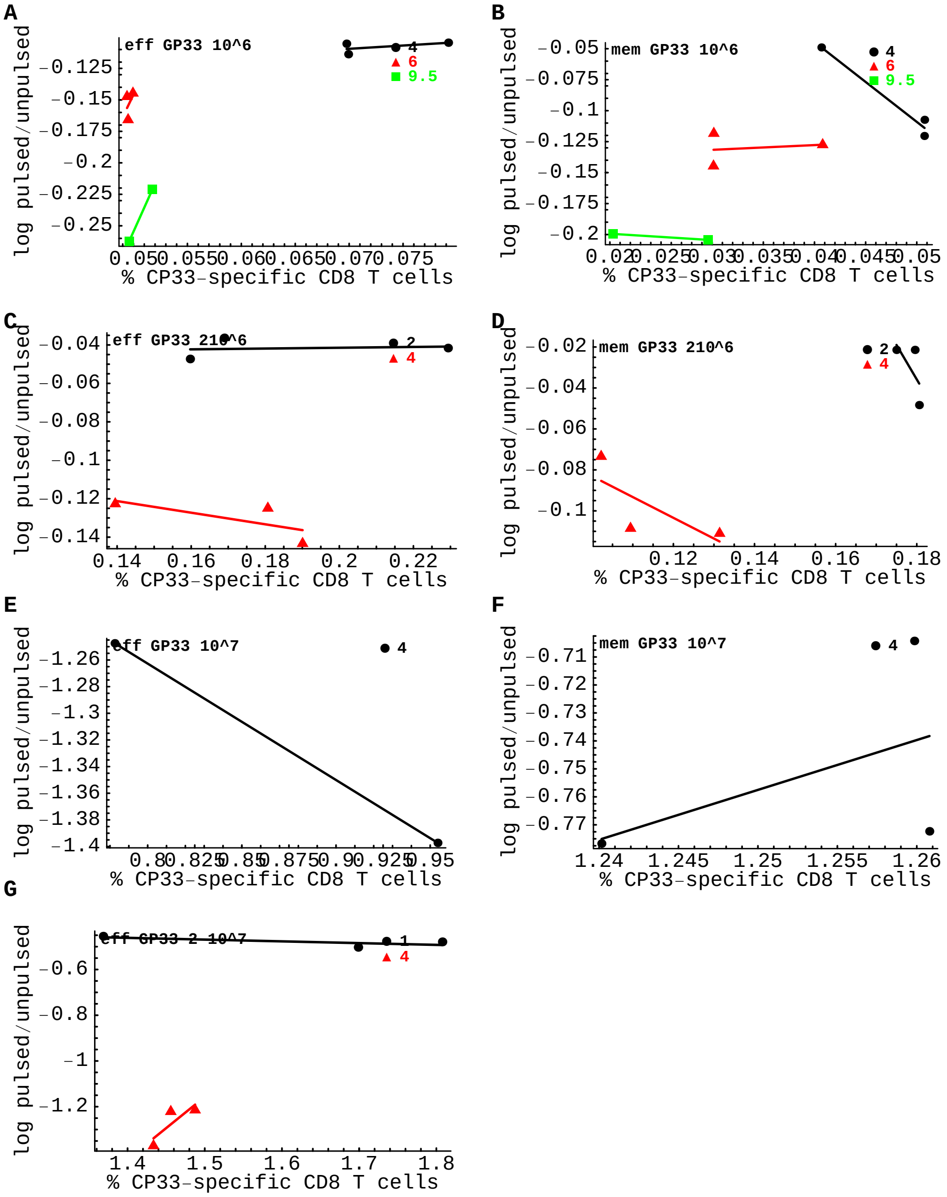

Figure S2: The observed correlation between the ratio of the frequency of GP33-pulsed and unpulsed targets and the frequency of GP33-specific $CD8^+$ T cells in the spleen at different time points after transfer in the adoptive transfer experiments. Each panel depicts identically treated mice having a particular number of effector cells transferred, the horizontal axis depicts the unintentional variation in the effector density in each group of mice. Lines show linear regressions through the various time points per mouse. If the unintended variation were real these regression lines should have negative slopes because pulsed target cells would be lost more rapidly in mice having more $CD8^+$ effector cells. Note that there is a relatively small variation in the measured frequency of epitope-specific $CD8^+$ T cells (as compared to variation in Fig. S1).
